# Posterior Precortical Vitreous Pocket in Stickler Syndrome: A Report of Two Cases

**DOI:** 10.7759/cureus.59633

**Published:** 2024-05-04

**Authors:** Tetsuhiro Nagashima, Hideo Akiyama, Kosuke Nakamura, Shunsuke Tokui, Keisuke Nitta

**Affiliations:** 1 Department of Ophthalmology, Gunma University Graduate School of Medicine, Maebashi, JPN

**Keywords:** posterior vitreous detachment (pvd), abnormalities of collagen and elastin, retinal detachment (rd), posterior precortical vitreous pocket, stickler syndrome

## Abstract

Stickler syndrome is a genetic disorder characterized by collagen abnormalities leading to various ocular manifestations, such as retinal detachment. We present two cases of siblings clinically diagnosed with Stickler syndrome who exhibited retinal detachment. Case 1, a seven-year-old girl, and case 2, her 14-year-old brother, both displayed severe myopia and other clinical signs consistent with Stickler syndrome. Despite their ages, neither case showed evidence of posterior precortical vitreous pocket (PPVP) on imaging or during surgical intervention. These findings suggest a potential relationship between collagen abnormalities and PPVP dysplasia in Stickler syndrome.

## Introduction

Stickler syndrome is a common disease, characterized by vitreous humor liquefaction and degeneration due to genetic mutations involved in collagen formation, which results in retinal detachment in childhood [1]. The posterior precortical vitreous pockets (PPVPs) emerged in front of the macula as a solitary space in early childhood. They first were narrow liquefied spaces anterior to the macula at age three and evolved into small boat-shaped spaces that gradually enlarged with age. The channels connecting the PPVPs and Cloquet's canal begin to form after age five and up until they approach almost adult size at around age eight [2]. ﻿The role of PPVPs in various vitreoretinal interface diseases, such as macular holes, epiretinal membranes, and proliferative diabetic retinopathy has been reported [2]. In this study, we report cases of retinal detachment in siblings with clinically diagnosed Stickler syndrome in which the presence of a PPVP, which would normally be identified at their age, could not be confirmed.

## Case presentation

Case 1

**Figure 1:**
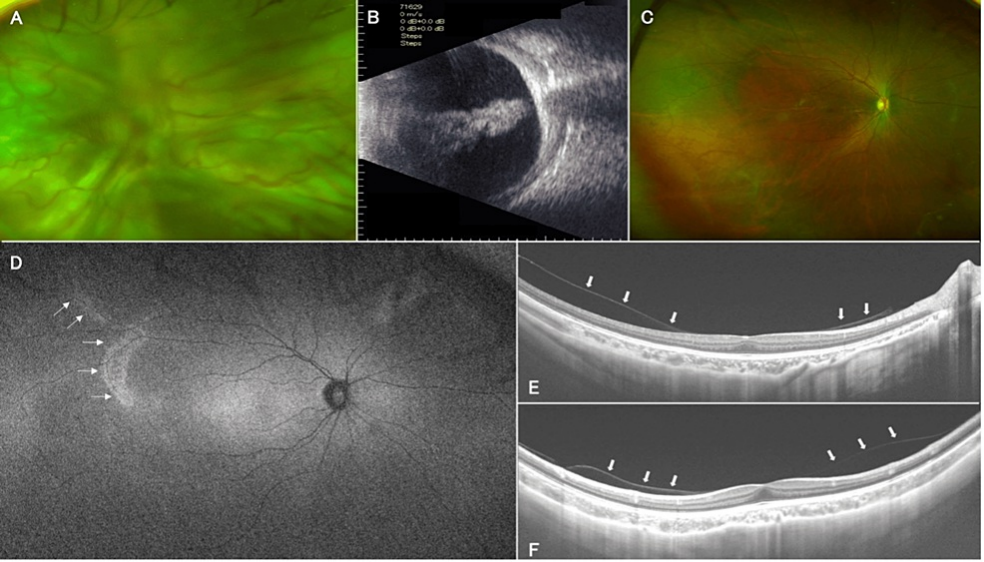
Ophthalmologic images of case 1. Ultra-widefield fundus photograph (A) and ultrasound examination (B) of the left eye show the presence of a funnel-shaped total retinal detachment that developed into severe proliferative vitreoretinopathy (grade CA). A color fundus photograph shows paravascular retinochoroidal atrophy of the right eye (C), which corresponds to the hyperfluorescence (arrow) observed in the fundus autofluorescence image (D). A horizontal (E) and vertical (F) swept-source optical coherence tomography (SS-OCT) B-scan through the fovea did not reveal any boat-shaped posterior precortical vitreous pocket but did show vitreoschisis with the posterior vitreous cortex splitting (arrow) into two layers in a parafoveal lesion.

A seven-year-old girl (younger sister) was found to have blurred vision during a physical examination at five years of age. She was prescribed glasses for amblyopia with a refraction of -14D in the right eye and -13D in the left eye. Her best-corrected visual acuity (BCVA) was 20/200 and 20/100 in the right and left eye, respectively. After the child’s mother noticed that her left eye was out of alignment, she visited a private clinic and was subsequently referred to our department due to finding retinal detachment in the left eye (Figures 1A, 1B).

At the initial visit, BCVA was 20/50, with light perception in the right and left eye, respectively. Intraocular pressure was 17 and 9 mmHg in the right and left eye, respectively. She had a total retinal detachment (grade CA) complicated by severe proliferative vitreoretinopathy (Figures 1A, 1B). However, after some discussions with her family, she decided to follow up afterward without treatment. While it was unclear when the girl's retinal detachment occurred, her medical history included surgery for a small jaw and cleft palate, along with a history of intubation management for Pierre Robin syndrome. Family history included severe myopia in her mother and a history of laser treatment for a retinal tear that occurred when the patient was in junior high school.

An ultra-widefield fundus autofluorescence photograph (Optos® 200Tx, Optos PLC, Dunfermline, United Kingdom) revealed hyperfluorescence corresponding to radial paravascular retinal degeneration (Figures 1C, 1D). This was clinically suspicious for Stickler syndrome [3]. In addition, swept-source optical coherence tomography (SS-OCT) (DRI OCT-1 Triton, Topcon Corp., Tokyo, Japan) did not clearly show any PPVP (Figures 1E, 1F).

Case 2

**Figure 2:**
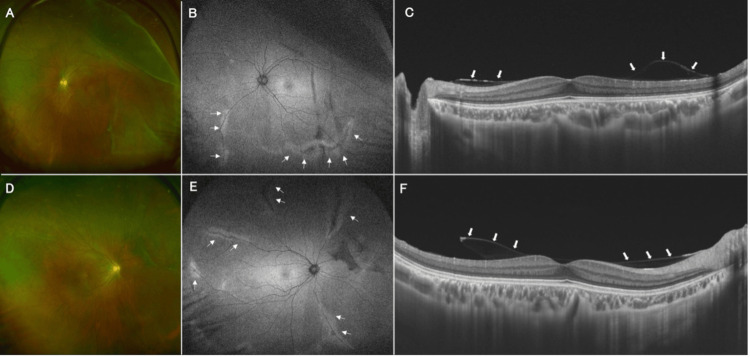
Ophthalmologic images of case 2. Ultra-widefield fundus photograph (A) shows retinal detachment accompanied by a superotemporal giant tear of the left eye. Ultra-widefield fundus photographs (A and D) also reveal paravascular retinochoroidal atrophy, which corresponded to the hyperfluorescence (arrow) observed in the fundus autofluorescence image (B and E). A horizontal swept-source optical coherence tomography (SS-OCT) B-scan through the fovea did not reveal any boat-shaped posterior precortical vitreous pocket (PPVP) of the right (C) and left eye (F), which was similar to the images observed for the patient’s younger sister.

A 14-year-old boy (older brother) was diagnosed with a retinal detachment two years after the same diagnosis in his sister, and he was referred to our department. At the initial visit, BCVA was 20/16 and 20/16 in the right and left eye, respectively. The refraction was -6.00 D spherical and −2.00 D cylindrical × 180 in the right eye and -5.50 D spherical and −2.50 D cylindrical × 5 in the left eye. No abnormalities were identified during the anterior examination. An ultra-widefield color fundus photograph (Optos® 200Tx, OPTOS PLC) revealed the presence of a superotemporal retinal tear and retinal detachment in the left eye (Figures 2A, 2D).

Fundus autofluorescence showed hyperfluorescence that corresponded to radial paravascular retinal degeneration in both eyes (Figures 2B, 2E). As shown in Figures 2C, 2F, SS-OCT did not determine the presence of any boat-shaped PPVP structure in either of his eyes, even though he was 14 years old.

**Figure 3:**
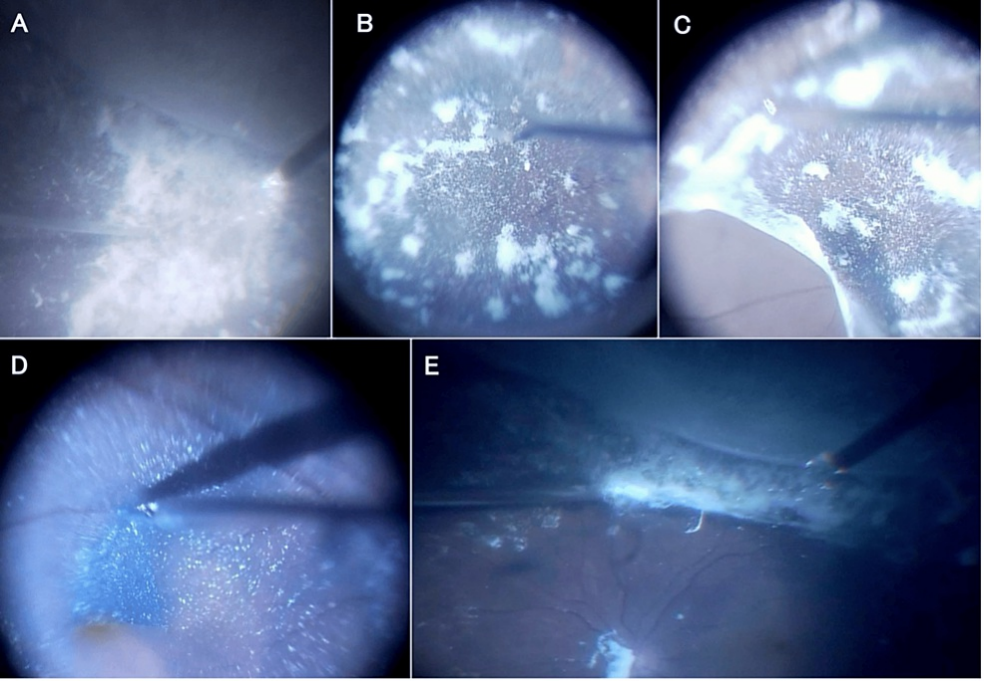
Intraoperative findings of case 2 during vitrectomy of the left eye. Vitreous staining with triamcinolone acetonide (TA) did not reveal any obvious posterior precortical vitreous pocket (PPVP) (A and B). The posterior wall of the premacular vitreous cortex, which was sticky and adherent to the surface of the retina, was peeled off using forceps to create the posterior vitreous detachment (C). Peeling of the internal limiting membrane (ILM) stained with brilliant blue G (BBG) was then carefully performed (D). The peeling of the vitreous cortex was then extended toward the periphery using a cutter (E).

During the immediate vitrectomy for the retinal detachment in the left eye, vitreous staining with triamcinolone acetonide (TA) did not reveal any obvious PPVP. Furthermore, there was no posterior vitreous detachment and a sticky vitreous cortex was adherent on the retina from the posterior pole to the periphery (Figures 3A, 3B).

The vitreous cortex of the posterior pole was carefully peeled with an extruder and forceps (Figure 3C), followed by peeling of the internal limiting membrane (Figure 3D). The peeling of the vitreous cortex was then extended toward the periphery using a cutter (Figure 3E).

The patient had a history of cleft palate and had previously undergone surgery for exudative otitis media associated with a buried ear. The patient was referred to our orthopedic department as the patient’s preoperative chest radiographs showed scoliosis and was suspected of having Stickler syndrome. The orthopedic radiographs also showed an enlarged transverse diameter of the proximal and distal femur, eversion of the hip joint, and flattening of the metacarpal head, which was indicative of osteogenesis imperfecta of the hand. Even though we did not perform a genetic evaluation, a clinical diagnosis of Stickler syndrome was made based on the other findings.

## Discussion

Stickler syndrome was first described in 1965 by Stickler [1] as an autosomal-dominant genetic disorder that is characterized by severe myopia, chorioretinal atrophy, retinal detachment, joint abnormalities, and deafness. Collagen plays an important role in maintaining the three-dimensional structure of the optical environment of the eye. Collagen molecules have a triple-helix structure in all tissues, including the skin, joints, and eyes, with the structure composed of three polypeptide chains. Currently, a total of 27 types of collagen have been identified, for which there have been 40 associated genes identified [3]. The collagen types present in cartilage and the vitreous body are types II, V, VI, IX, and XXVII [4]. Stickler syndrome is clinically classified into three types: type I, in which membranous structures are seen in the vitreous body; type II, in which bead-like structures are seen; and type III, in which no ocular findings are seen. In our two present cases, both siblings had severe myopia, and the mother had a history of treatment for retinal tear. Thus, this suggests that the mother may have had a genetic abnormality for an autosomal dominant genetic disorder that causes cleft palate and vitreous collagen abnormalities. Then we consulted specialists in pediatric orthopedics for case 2. The X-ray findings of long canal bone changes and epiphyseal dysplasia of the hand also led them to strongly suggest that the patient had Stickler syndrome. Case 2 also had a cleft palate and scoliosis, while case 1 had a history of exudative otitis media. They were also considered for genetic testing, but this was not done due to lack of consent. According to the scoring system reported by Peter S Rose et al. [5], cleft palate, vitreous or retinal degeneration, and high-tone hearing loss are scored 2, while Pierre Robin sequence and hypermobility eardrum, orthopedic abnormalities, namely, each femoral head failure, radiographically demonstrated osteoarthritis under 40 years of age, and scoliosis are scored 1. Then Stickler syndrome is clinically diagnosed if the score is more than 5. Case 1 scores 5 and case 2 scores 8, so both cases are clinically diagnosed as Stickler syndrome.

Case 1 had a total retinal detachment (grade CA) that was complicated by severe proliferative vitreoretinopathy. After some discussions with her family, it was decided to follow up without treatment. In contrast, case 2 underwent immediate vitrectomy. Although the long-established scleral buckling techniques are absolutely indicated for rhegmatogenous retinal detachment without posterior vitreous detachment (PVD), Abeysiri et al. [6] previously reported that vitrectomy (84.2%) has a higher reinstatement rate as compared to scleral entropion (66.7%) for detachment associated with Stickler syndrome. Thus, based on these findings, we performed a vitrectomy for this case. During the vitrectomy, the vitreous was visualized by spraying TA into the vitreous cavity [7], which was triggered by aspirating the vitreous cortex of the posterior pole with an extrude, thereby causing PVD to the periphery through the use of a vitreous cutter. When PVD has not occurred and the anterior border of the PPVP is broken by core vitrectomy, subsequent application of TA should stain the posterior and the lateral border of the PPVP. However, in case 2, the lateral border of the PPVP, which arises from the retinal surface, could not be identified no matter how much peripheral vitreoretinal border was vitrectomized, indicating that the PPVP was not present.

It has been previously reported that the PPVP can be observed starting from around three years of age and up until it approaches almost adult size at around eight years of age [8]. In our present report, preoperative SS-OCT images showed that there was an absence of a clear PPVP in both cases. The younger sister was seven years old, while the older brother was 14 years old. Thus, in both of these cases, the formation of a PPVP close to the size seen in healthy adults should have been observed. However, there was no PPVP observed in either of the two cases. In addition to the images from the SS-OCT, the outer margin of the PPVP was not observed during the surgical procedure in the older brother. Therefore, it is speculated that there may be a relationship between the vitreous liquefaction associated with collagen abnormalities and PPVP dysplasia. Chen et al. reported giant premacular bursas (i.e., giant PPVPs) in two cases of Stickler syndrome [9]. As we have previously reported, the PPVP appeared as a narrow liquefied space in front of the fovea at age three. Subsequently, the PPVP enlarged and the anterior border became well-demarcated from the age of four years [8]. To call the premacular bursa a PPVP, it needs to visualize an anterior border. However, the anterior border of what they call the giant premacular bursa was not visualized since it was extended beyond the scan depth and width of the SS-OCT they used. So it is unclear if the giant premacular bursa that Chen et al. reported was a true pocket. We speculate that the giant premacular bursa that Chen et al. reported was liquid vitreous rather than a giant PPVP. In this view, their optical coherence tomography (OCT) findings are consistent with our OCT and surgical findings.

## Conclusions

Two siblings presented with retinal detachment and were clinically diagnosed with Stickler syndrome, a collagen disorder. Both cases lacked a PPVP, which would normally be present at their age. It is speculated that there may be a relationship between the vitreous liquefaction associated with collagen abnormalities and PPVP dysplasia in Stickler syndrome. This finding potentially contributes to a better understanding of the pathogenesis of Stickler syndrome and vitreoretinal diseases.
